# The application of mixed reality technique in oromaxillo-facial reconstruction with the perforator flap for malignant tumor patients

**DOI:** 10.3389/fonc.2024.1437598

**Published:** 2024-07-19

**Authors:** Yixiu Liu, Jian Wu, Daide Liu, Dalan Xiang, Xiaoyue Wu, Ting Wang

**Affiliations:** ^1^ Head and Neck Surgery, Chongqing University Cancer Hospital, Chongqing, China; ^2^ Department of Surgery, People’s Hospital of Shizhu, Chongqing, China; ^3^ Internal Medicine-Oncology, Chongqing University Cancer Hospital, Chongqing, China

**Keywords:** mixed reality, computed tomography angiography, perforator, oromaxillo-facial reconstruction, malignant tumors, cancer care

## Abstract

**Objectives:**

The integration of quantitative imaging techniques such as computed tomography (CT) and magnetic resonance imaging (MRI) with mixed reality (MR) technology holds promise for enhancing the diagnosis, prognosis, and treatment monitoring of cancer. This study compares the characteristics and effects of MR and color Doppler ultrasound (CDU) in the localization of perforator blood vessels in the lower extremities.

**Methods:**

Two techniques were used to locate the perforator vessels in 40 cases of maxillofacial defect repair using perforator flaps from the lower extremities. The number of perforator vessels located in the flap area and the actual number of perforator vessels explored during the surgery were recorded. The recognition rate was calculated and the operation time and blood loss were recorded for each case.

**Results:**

The recognition rates of MR technology and CDU in perforating vessels of the lower limbs were 93.9% and 97.2%, respectively (*p >* 0.05). The operation time was 52-74 minutes, 65-88 minutes (*p >* 0.05). The average bleeding volumes were 24 and 56 ml (*p <* 0.05), respectively. All perforator flaps were alive. One flap had a crisis and recovered after emergency exploratory treatment. Thirty donor sites of the lower extremities were directly sutured, and wounds were closed by abdominal skin grafting in 10 cases.

**Conclusion:**

MR technology for successfully identifying perforator vessels can shorten the operation time, reduce the amount of bleeding in the donor site, and reduce trauma to the donor site.

## 1 Introduction

Koshima and Soeda introduced the concept of the perforator flap in 1989, which differs from the conventional flap, which requires dissection from the main vessel to the distal branch vessels (1). Instead, the perforator flap utilizes retrodissection from the peripheral perforator vessels to the source vessel. By identifying the perforator vessels in the designated area, the flap can be harvested via dissection of the perforator vessel (2). This approach allows for successful outcomes even if the surgeon is not entirely familiar with the tissue structure in that region. Furthermore, the perforator flap method offers several advantages, including flexibility, ease of transfer, minimal damage to donor sites, and clinical reliability and efficacy (3–6). As a result, the perforator flap has become the preferred choice for repairing soft tissue defects and reconstructing the jaws in head, neck, and maxillofacial surgeries. However, the accuracy and predictability of clinical applications are limited by differences in the location, origin, and course of perforator vessels among individuals, which can affect the final repair outcome. Therefore, accurate identification of perforator vessels and appropriate flap design are crucial clinical considerations that must be addressed (7, 8).

The mixed reality (MR) technique is lately introduced that leverages computed tomography (CT) and magnetic resonance imaging (MRI) data in a software workstation to generate a 3D model, allowing it to be downloaded to a head-mounted holographic display (9). In clinical settings, holographic displays are used by operators to project the model onto a patient’s body surface and subsequently match it to the relevant organ based on locators or fixed anatomical markers embedded in the tissue. The positioning error between the virtual model and patient is less than 1 mm (10), with negligible discrepancies of 2-3mm observed in the process of surgical anatomy (11). In this particular investigation, computed tomography angiography (CTA) data were used to construct a 3D model, and MR technology was used to facilitate real-time location of the lower limb perforator vessels during surgical procedures. In this study, we compared this new technique with color Doppler ultrasound (CDU) and evaluated its effects on the preparation and harvest of perforator flaps. This study introduces a novel and efficient approach for conducting lower limb perforator flap surgery, offering significant support for the application of mixed reality technology in the medical field. Our findings provide important insights for enhancing surgical outcomes, mitigating intraoperative risks for patients, and elevating overall medical quality. These discoveries hold promising implications for advancing the utilization of mixed reality technology in medicine.

## 2 Materials and methods

### 2.1 Clinical data

Our study included 40 patients (29 males and 11 females) aged between 32 and 75 years of age (mean age, 57.3 years) who underwent harvesting perforator flaps in the lower extremities at the Chongqing University Cancer Hospital between January 2019 and September 2020. These patients were randomly assigned to two groups of 20 patients each: the experimental group, which used MR technology with CTA data to locate perforator vessels, and the control group, which used CDU. All perforator flap surgeries were performed by the same surgeon. The inclusion criteria were as follows: (A) patients with malignant tumors in the oral and maxillofacial regions and clinically diagnosed at the middle or advanced stage; and (B) patients who were unable to undergo forearm free-flap reconstruction due to extensive tissue defects resulting from tumor excision. The exclusion criteria were as follows: (A) patients who had small primary lesion defects that could be sutured or repaired with a free-flap forearm reconstruction; (B) patients who had previous surgeries or trauma in both lower limbs; (C) patients who were unable to accept general anesthesia and surgeries for various reasons.

### 2.2 Lower limb CTA examinations of the experimental group

The scan was performed using a dual-source CT system (Siemens Somatom Drive; Siemens, Erlangen, Germany). Patients were scanned in a supine position with their feet first and their arms placed higher than the head in the same position as the surgery. The iodinated contrast agent iopromide 370 was injected at 4.0 ml/s for a total volume of approximately 90 ml. The arterial phase was triggered by monitoring the femoral artery with a trigger threshold of 100 HU, and the delay phase was 10 s. After the scanning was completed, the original image data of 1 mm layer thickness without horizontal axis spacing were transmitted to the workstation, and the image was post-processed by multi-plane reconstruction, maximum density projection, volume reconstruction, surface reconstruction, and other technologies.

### 2.3 3D reconstruction and import of experimental CTA data

We imported the obtained CTA digital imaging and communications in medicine (DICOM) data into a software workstation for staging and reconstruction of the original data. The selected region was used to determine the threshold value and generate the masking-out of the reconstructed area to construct a 3D model. A preliminary 3D model was then established and further edited by smoothing the soft tissue, bone tissue, and blood vessels. The resulting model was optimized by refining the triangular surfaces, removing any protrusions, and hollowing out blood vessels or cavity organs. The boundary contour was adjusted, and a 3D model was prepared. The 3D reconstruction model was imported into a head-mounted holographic display via the internet. The production and import of all the data were completed by the same technician.

### 2.4 The CDU examination and vascular localization towards the lower extremities of control group

Ultrasonic inspection was performed using a Philips Blood Vessel Detector (Philips, Best, Netherlands) at a 7.5 MHz frequency. The patients were instructed to adopt the corresponding posture according to the operator. In addition to ultrasonic measurement of perforator vessels in the donor sites of the lower limbs, the course, diameter, and blood flow dynamics of the perforator vessels were recorded. Marks were also made on the body surface projections of the perforator vessels. Both measurements and CDU marks were performed by the same medical technician.

### 2.5 Surgical procedures

The superior thigh of the perforator artery was selected as the donor site. In the experimental group, the flaps were designed with the perforating point of the perforator vessel as the center point, as determined by a head-mounted holographic display according to the scope, shape, and size of the defect area. In the control group, the flaps were designed with the perforating point of the vessel as the center point, as determined by CDU prior to surgery. After dissection of the skin and subcutaneous adipose tissue, the perforator vessels below the surface of the fascia lata were identified directly from the front to the rear. The perforator vessels were well preserved after dissection. After preparation of the tissue flaps was completed, the pedicles were dissected, and blood vessels were transected and ligated near the proximal end for anastomosis with the blood vessels at the primary lesion site. The defect in the donor area of the lower-extremity epidermis was closed or sutured with a skin graft.

### 2.6 Recording of measurement indicators

In this section, we validate the accuracy of the two techniques for localizing perforator vessels applied in the experimental and control groups. In addition, the number of localized perforator vessels and the actual perforator vessels within the flap area were recorded, and the recognition rate was calculated (recognition rate = number of located vessels/number of actual perforator vessels). The number of cases of direct closure suture or skin graft suture in the experimental and control groups, the time of completion of each operation, and blood loss volume in each group were also recorded.

### 2.7 Statistical analysis

The obtained data were statistically processed by means of SPSS statistical software.

## 3 Results

### 3.1 Lower limb perforator flap surgery outcomes and comparative analysis of MR and CDU techniques

Free perforator flaps from the lower limbs were successfully obtained in all 40 patients who underwent surgery, and all the flaps survived after surgery. One patient in the experimental group needed urgent re-exploration and salvage of the flap 4 h after surgery and succeeded in treating flap complications. Among the 20 patients in the experimental group, 18 underwent anterolateral thigh flap reconstruction, 2 underwent fibula osteocutaneous flap repair, 16 underwent direct suture closure in the lower limbs, and 4 underwent skin graft suturing. The operation time ranged from to 52-74 min, and the average blood loss volume was 24 ml. Among the 20 patients in the control group, anterolateral thigh flaps for reconstruction were adopted in 17 cases, fibula osteocutaneous flap repair was used in three cases, direct suture closure in the lower limbs in 14 cases, and skin graft in six cases. The operation time ranged from to 65-88 min, and the average blood loss was 56 ml. The identification rates of MR and CDU in perforator vessels of the lower limbs were 93.9% (31/33) and 97.2% (35/36), respectively. Statistical analyses showed that there was no significant difference in operation time between the two groups (*p >* 0.05). The recognition rate of MR technology is slightly lower than that of CDU, but there is no significant difference between the two groups (*p >* 0.05) (details are presented in **Table 1**).

**Table 1:** Flap details of the experimental and control groups.

	Number (Cases)	Age (Y/O.)	Repair method (Cases)	Donor site management (Cases)	Operation time (min)	Average blood loss (mL)	Recognition rate (%)
Experiment	20	32-68	Anterolateral thigh flaps (18)	Direct suture (16)	52-74	24	93.9
			Fibula osteocutaneous flaps (2)	Skin graft repair (4)			
Control	20	35-75	Anterolateral thigh flaps (17)	Direct suture (14)	65-88	56	97.2
			Fibula osteocutaneous flaps (3)	Skin graft repair (6)			

### 3.2 Case presentation

**Figure 1:**
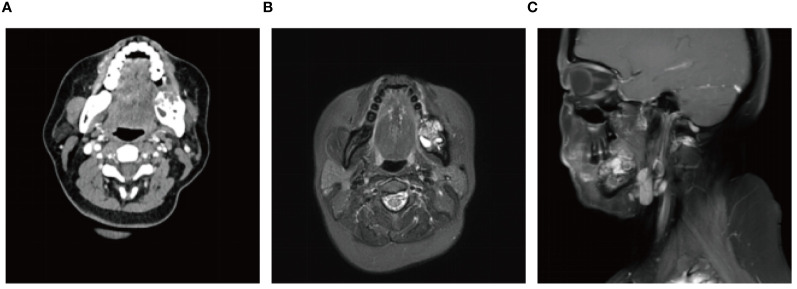
Preoperative maxillofacial imaging. **(A)** Cross-section plane of the preoperative computed tomography scan; **(B)** Cross-section plane of preoperative magnetic resonance; **(C)** Coronal plane of preoperative magnetic resonance.

**Figure 2:**
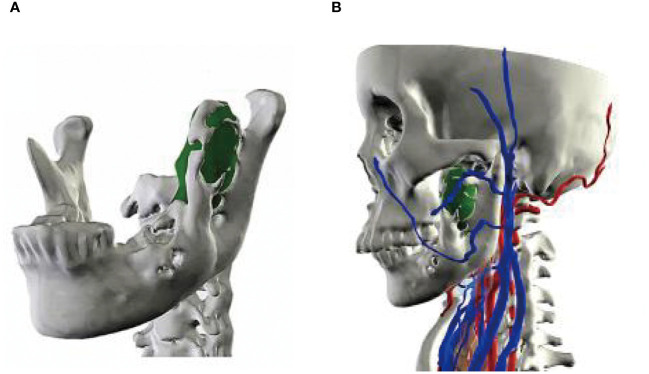
Maxillofacial 3D model. **(A, B)** Lateral views of the model.

**Figure 3:**
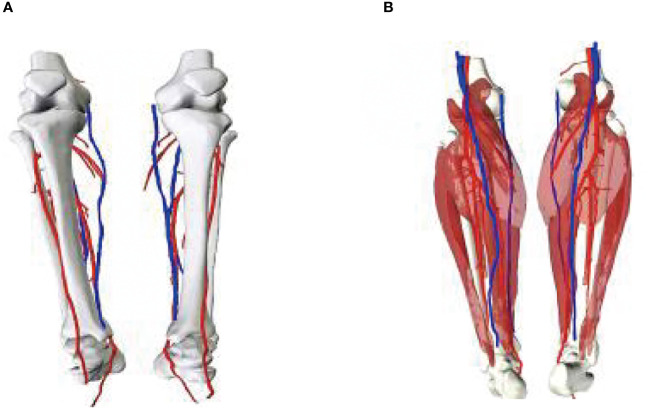
3D models of blood vessels in lower extremities. **(A)** Front; **(B)** Posterior views of the model.

**Figure 4:**
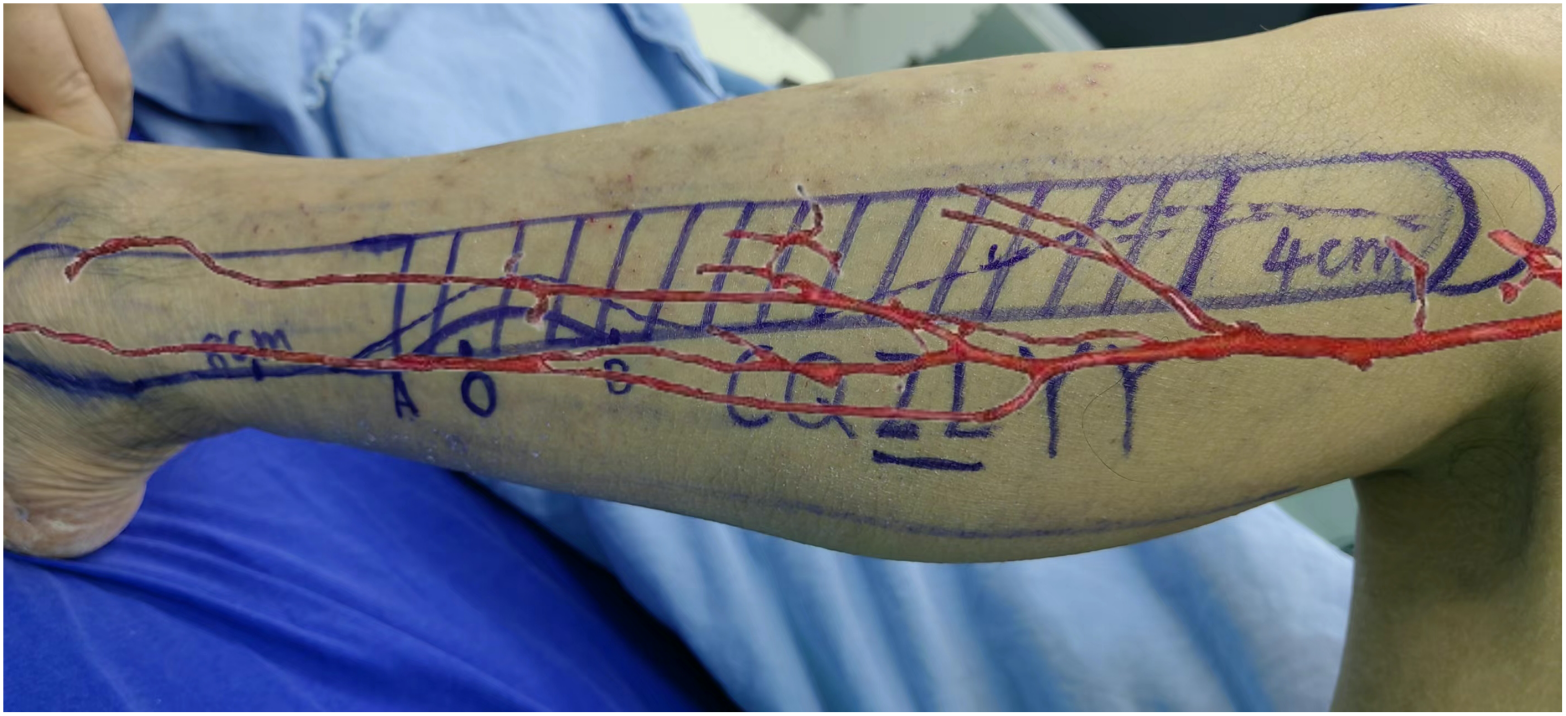
Intraoperative schematic diagram of mixed reality technology.

**Figure 5:**
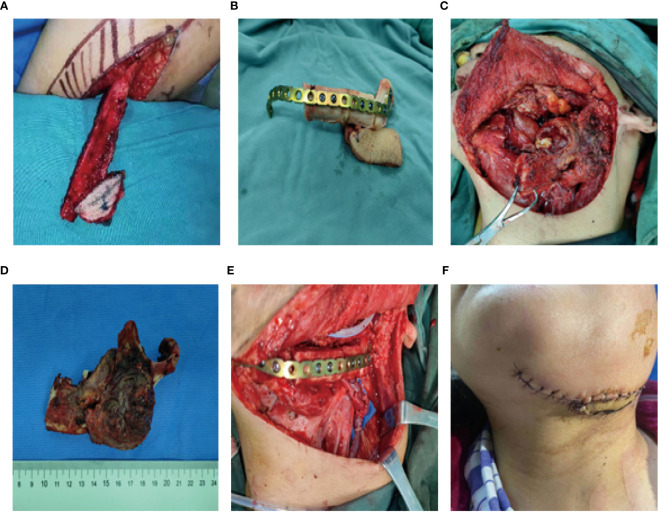
Repair and reconstruction of peroneal musculocutaneous flap. **(A–E)** Intraoperative reconstruction with fibula musculocutaneous flap; **(F)** Reconstruction outcome after the surgery.

**Figure 6:**
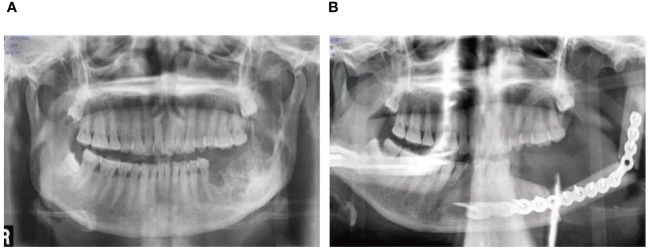
Repair and reconstruction of peroneal musculocutaneous flap. **(A)** Panoramic radiograph before treatment; **(B)** Panoramic radiograph after treatment.

A 56-year-old female underwent surgery for ameloblastoma in the left mandible 2 years previously. Specialist examination revealed that the patient had swelling on the left face and neoplasm in the area surrounding the left mandibular angle and ramus, approximately 5.0 × 4.0 cm measuring in size. The patient also had a mild limitation in mouth opening, and 36, 37, and 38 were not detected in the oral cavity. According to the patient’s previous medical records and consultation report from the pathology department of our hospital, the pathological diagnosis of the patient was confirmed as ameloblastoma (**Figure 1**). The patient was diagnosed with ameloblastoma upon admission and scheduled to undergo partial mandibular resection and fibular osteocutaneous flap repair. She underwent CTA examination of the maxillofacial and the left calf prior to surgery. The obtained CTA data were imported into a software workstation for 3D reconstruction and subsequently downloaded onto a holographic display (**Figures 2**, **3**). During the operation, the left calf was automatically positioned according to the holographic display, and the perforator vessels were dissected in real-time (**Figure 4**). Fibular osteocutaneous flaps were prepared to repair the mandible, and direct sutures were placed at the donor site (**Figures 5**, **6**). After the operation, the flap fully survived, and during the 6-month follow-up, the patient expressed satisfaction with the reconstructive outcome.

## 4 Discussion

Since the 1990s, various techniques have been established to evaluate perforator vessel characteristics. Of these methods, the application of CTA and CDU is the most extensive (12, 13), but their effectiveness remains controversial. Many scholars believe that CTA is the gold standard for locating blood vessels (14), as it can precisely identify the course of vessels in muscles (15, 16) and shorten the operation time (17, 18). However, some scholars (19) also believe that CDU is superior to CTA in terms of the radiation dose and localization of superficial blood vessels. Currently, there is no consensus regarding the effectiveness of these two methods reported in literature. However, a common problem in clinical practice is that neither CTA nor CDU can provide real-time intraoperative guidance for perforator vessel localization. The 2D images from CDU only capture the superficial portion of perforator vessels on the body surface, lacking information on the 3D structural course of the vascular bundles. 3D CTA images require surgeons to match the reconstructed images with the actual surgical area during the operation based on their experience, which is time-consuming (20). Meanwhile, leaving the surgical field for a surgeon to view CTA and CDU images on the screen poses potential risks, including failure to promptly detect bleeding at the operative site or dropped instruments (21, 22).

Contextually, an ideal surgical navigation system should possess the following characteristics (23): (A) excessive additional work should not be introduced into the surgical process; (B) excessive invasive procedures should be avoided; and (C) rapid, real-time localization should be achieved and maintained in a sterile manner. Despite being a research hotspot in academia, current solutions for surgical navigation systems cannot meet the aforementioned requirements and, consequently, hinder the clinical application of navigation matching.

MR technology is a novel technique that has gained popularity in recent years, and it offers multiple advantages for its applications in the medical field (10, 24, 25): (A) It provides surgeons with intuitive and real-time imaging information to view both deep and superficial anatomical structures, lowering the difficulty in identifying tissue structures; (B) MR technology can superimpose images in real time onto the patient’s anatomical structure and guide surgery using virtual 3D visualization information, with great convenience in operation; (C) for tumor patients, MR technology can design tumor resection margins in advance, avoiding insufficient resection during surgery, which affects prognosis; (D) Through WIFI, MR technology transmits 3D models and matching images of patients to experts for remote communication and instruction during surgery; and (E) Compared with traditional positioning methods, surgeons can independently use HoloLens to project the 3D model of perforating vessels, touch and manipulate virtual objects, adjust the position, angle and scale of the 3D model, and overlap the 3D model on the human body to understand the shape and distribution of blood vessels and protect them during the flap preparation process, achieving aseptic operation.

**Figure 7:**
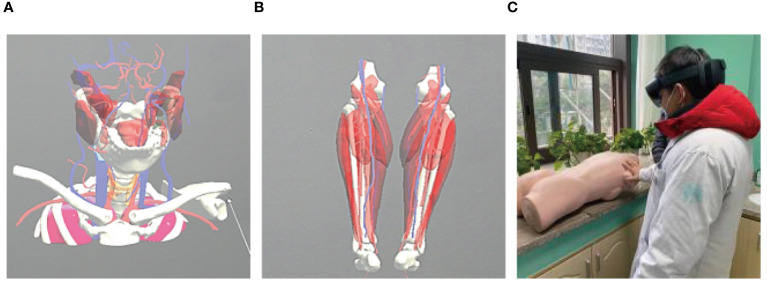
Application of mixed reality technology in teaching. **(A, B)** Video demonstration in teaching; **(C)** Teaching practice with holographic display.

**Figure 8:**
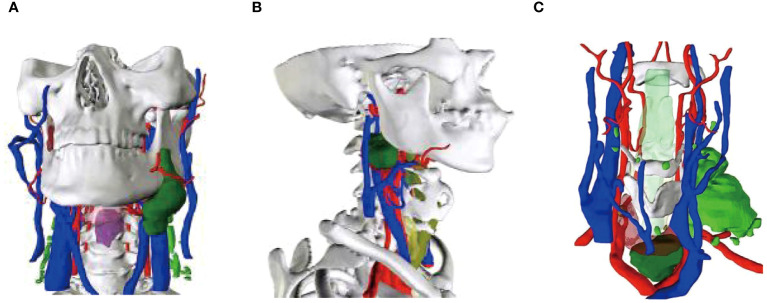
Mixed reality technology to rebuild the primary focus. **(A)** Buccal mucosa carcinoma reconstruction; **(B)** Oropharyngeal carcinoma reconstruction; **(C)** Thyroid malignancy reconstruction.

After applying MR technology in the field of orthopedics, Lee et al. (26) demonstrated that displaying the anatomical structure of skin-covered areas aids surgeons in rapid localization during screw placement and reduces surgical risks, thus improving surgical accuracy. Shi et al. (27) were the first to apply MR technology in hepatectomy and achieved accurate matching between a 3D hologram model and the target organs. Thus, MR technology can be combined with other clinical treatment techniques. For example, when combined with a da Vinci robotic system, it can minimize trauma and surgical complications while achieving the goal of curing lesions (28, 29). Although MR technology has been applied in orthopedics, hepatobiliary surgery, and neurosurgery (30–32), there are relatively few reports on its application in the field of oral and maxillofacial surgery (33–35). Therefore, in the early stages of the study, the author first attempted to apply MR technology to clinical teaching and doctor-patient communication, which achieved good results and accumulated rich experience (**Figure 7**). In this study, we used MR technology to perform 3D reconstruction of the maxillofacial region in 40 patients with malignant oral tumors. We found that the reconstructed 3D models were accurate and intuitive, and could be overlaid on the surgical site in real time with high precision. Compared to traditional imaging techniques, MR technology allows for easier understanding of organ anatomy, tumor shape, and location and expands the previously limited view of maxillofacial surgery in terms of depth and breadth, reducing judgment time and mental workload. In this study, the 3D reconstruction of the mandible in five patients was more distinct in terms of three-dimensional sense and boundary level compared to the reconstruction of soft tissues (**Figure 8**).

Recently, there has been an increasing interest among scholars in utilizing MR technology for the vascular localization of perforator flaps. This technology enables the observation of the origin, course, branching, and distribution of perforator vessels in 3D space and facilitates the reconstruction of precise 3D visualization models of perforator flap vessels. Bosc (36) employed MR technology to locate the perforator vessels in the lower abdomen for breast reconstruction. CTA data are reconstructed and imported into a holographic display, which allows the automatic matching of skin locators or anatomical structures during surgery. As a result, surgeons are able to perform a visual operation during the reconstruction process without having to direct their gaze towards a distant screen, available for a “fluoroscopic” view of the blood vessels. Pereira (37) utilized MR technology to anatomically locate blood vessels in the groin area of 60 patients and found that the positions of all vessels and lymph nodes corresponded to the actual operative location. MR technology can accurately locate the position of blood vessels and lymph nodes in the groin area, reducing flap harvesting time by 20% compared to traditional methods. In this study, we performed CTA examinations of the lower limbs and created a 3D model from the acquired data, which was then imported into a head-mounted holographic display. With the help of the display, the perforator vessels can be automatically located without relying on experience or spending extra time. Reverse dissection was performed on the basis of the course of the vessels. The time required for flap harvest was approximately 52-74 minutes, saving about 20% of the time (65-88 minutes) required by traditional methods. The results of this study are consistent with those of Pereira et al., but the difference was not statistically significant. The head-mounted display used was Microsoft HoloLens, which is comfortable and lightweight. However, long-time wearing of this device may cause a sense of dizziness, which may be related to the LED lighting and video sensor flicker of the head-mounted holographic display. This is consistent with researchers in other countries who believe that no fatigue or pain is associated with prolonged device use (24, 38).

In clinical practice in our hospital, ALT free flap surgery usually uses CDU for vascular localization, and CTA is only used in a small number of patients. However, although CDU is simple and low-cost for vascular localization, it has a large error. Therefore, the original intention of this study was to explore new ways to locate blood vessels. A large number of clinical studies have shown that the accuracy of CTA in vascular localization is higher than that of CDU (39), and it is known as the “gold standard” for vascular localization (40). Currently, there are no relevant literature reports on the clinical application of MR-superimposed CTA data for vascular identification. This study used MR technology to locate blood vessels based on CTA data. Although it increased costs and made the surgical process relatively complicated to a certain extent, it achieved three-dimensional visualization of perforating vessels, which can intuitively understand the shape and distribution of perforating vessels and the location of the exit point, which is conducive to preoperative flap design, intraoperative protection of perforating vessels and exit points, and reducing the possibility of flap crisis. Based on this, we believe that the use of MR based on CTA data in ALT has both advantages and disadvantages. The advantage is that it can achieve three-dimensional visualization of perforating vessels, which is conducive to preoperative flap design, reduces the possibility of flap crisis, and reduces the actual flap production time. However, the disadvantage is that the process is relatively complicated and the cost is increased.

Moreover, this study validated the accuracy of MR technology and CDU in identifying lower limb vessels by anatomical dissection. The results showed that the identification rate of MR technology was 93.9%, which was slightly lower than that of the conventional CDU (97.2%). We believe this may be explained by the following reasons: (A) small sample size; (B) new technologies may have inherent positioning errors (MR errors plus CTA errors); (C) the curved contours of the lower limbs may cause vessel displacement relative to the skin, leading to measurement errors; and (D) variations in adipose tissue thickness among cases may also have contributed to errors. MR technology can provide complete perforator information in patients with a thick adipose layer. However, in areas where the adipose layer is thin, the display of the end of the perforating vessels may be unclear because of the influence of CTA data on the MR technology. Although MR technology had a lower identification rate than traditional positioning methods in this study, it can provide surgeons with 3D courses of perforating vessels in practical operations, achieving the effect of “fluoroscopic” and precise anatomy once identified. Meanwhile, the average blood loss during the dissection of perforator flaps was 24 ml using MR technology and 56 ml in the control group, and the difference between the two was statistically significant. Hence, we assumed that the successful identification of perforating vessels using MR technology may result in reduced flap harvesting time, decreased blood loss, fewer postoperative complications, and eventually, benefit patients.

There are many types of free flaps available to head and neck reconstructive surgeons (41), the most commonly used of which are the radial forearm flap (RFF) and the anterolateral thigh flap (ALT) (42). Since the 1980s, RFF and ALT have been widely recognized as versatile and reliable free perforator flaps (43, 44). With significant advances in microsurgical technology, the success rate of free flap transplantation has increased to more than 90% in most published case studies (45, 46). Las, D.E (47). counted 1530 free flaps in 1247 patients, and the incidence of partial and total flap necrosis was 5.5% and 4.4%, respectively. WZhou et al. (48) included 881 flap transplants of the head and neck. Only 26 of the 881 flaps failed (2.9%). In this study, age, diabetes, history of lateral neck surgery, donor site, selection of recipient vein, and postoperative anticoagulation were not related to the outcome of free flaps, which were mainly affected by preoperative radiotherapy. At the same time, Ranganath (49) conducted an electronic search using PubMed, EMBASE, and the Medline Database of Systematic Reviews (CDSR), including all papers published between 2000 and 2022, and combined the following keywords: (RFF), (ALT). The final meta-analysis included 16 studies (50–65) that evaluated flap success rates, with a success rate of 98.3% (460/468) in ALT patients and 97.3% (476/489) in RFF patients. In this study, one of the 40 perforator flaps had a flap crisis 24 hours after surgery. After timely inspection by the nurse and timely vascular exploration in the operating room by the doctor, the flap eventually survived. However, in the treatment of our hospital, there are still cases of flap necrosis, mainly in patients who underwent surgery after radiotherapy. The overall flap success rate is not much different from the success rate reported by the researchers. In the future work, we will further statistically analyze the data of the flap success rate in our hospital.

In summary, MR technology has the potential to be applied in the repair and reconstruction of oral and maxillofacial defects by using flaps. This technology has the capability to successfully identify perforator vessels, which results in reduced blood loss and shorter surgical time, and may emerge as a novel auxiliary tool for future microsurgery. Despite the promising results, several limitations should be acknowledged. Firstly, the sample size in our study was relatively small, which may limit the generalizability of our findings. Additionally, the retrospective nature of the study design and the lack of a randomized control trial may introduce bias and confounding variables. Moreover, the study focused exclusively on lower limb perforator flap surgery, and the applicability of MR technology in other surgical contexts remains to be explored. Future research with larger sample sizes and prospective study designs is warranted to further validate the utility of MR technology in reconstructive surgery and to address these limitations.

## Data availability statement

The original contributions presented in the study are included in the article/supplementary material, further inquiries can be directed to the corresponding author/s.

## Ethics statement

This clinical trial has been conducted in accordance with the principles of the Declaration of Helsinki and has received ethical approval from the Ethics Committee of Chongqing University Cancer Hospital (No. CZLS2021177-A). All participants provided informed consent before participating in the study. The study design, procedures, risks, and benefits have been thoroughly explained to the participants. Participants have the right to withdraw from the study at any time without any consequences. Confidentiality of all participant information is strictly maintained throughout the study. The studies were conducted in accordance with the local legislation and institutional requirements. The participants provided their written informed consent to participate in this study. Written informed consent was obtained from the individual(s) for the publication of any potentially identifiable images or data included in this article.

## Author contributions

YL: Conceptualization, Investigation, Project administration, Visualization, Writing – original draft, Writing – review & editing. JW: Conceptualization, Funding acquisition, Investigation, Writing – original draft, Writing – review & editing. DL: Data curation, Methodology, Writing – original draft. DX: Software, Writing – original draft. XW: Supervision, Writing – review & editing. TW: Supervision, Writing – review & editing.
